# Knowledge on HIV and its association with sexual behavior among adolescents in Kampala, Uganda

**DOI:** 10.4314/ahs.v25i2.28

**Published:** 2025-06

**Authors:** Alice Nakato, Joan Nakayaga Kalyango, Caroline Makoha, Phillip Orishaba, Apolo Ayebale, Sabrina Bakeera Kitaka

**Affiliations:** 1 Makerere University School of Medicine, Clinical Epidemiology and Biostatistics Unit; 2 Uganda Christian University, School of Medicine, Department of Research; 3 Makerere University School of Medicine, Department of Pharmacy; 4 Makerere University School of Medicine, Department of Paediatrics and Child Health

**Keywords:** Adolescent, HIV, Sexual Behavior

## Abstract

**Background:**

Despite several efforts to educate adolescents about HIV/AIDS in Uganda, many still engage in unsafe behaviors, putting them at risk of HIV infection.

**Objective:**

We aimed to assess the level of HIV knowledge and associated factors and establish association with sexual behavior among 13 to 19-year-old adolescents living in an urban division in Kampala district.

**Methods:**

We conducted a cross-sectional study among 976 adolescents selected through multistage sampling and administered one pretested questionnaire to collect data. We analyzed the data using Stata version 14.0.

**Results:**

Knowledge on HIV was moderate (mean score = 11.30, SD= 2.91), and it was not associated with sexual behavior (OR= 0.97, p-value = 0.565). HIV knowledge was associated with age, school status, and knowledge of HIV status.

**Conclusions:**

Knowing about HIV did not imply engaging in safe sex practices. There is, therefore, a need to encourage adolescents to adopt safe sexual behavior in the fight against HIV.

## Introduction

HIV continues to be a major problem of public health concern in the world today. In 2017, Uganda had 1.3 million people living with HIV^1^. According to the 2019 Uganda Population-based HIV Impact Assessment (UP-HIA) report, HIV was more prevalent in urban areas, where 16.6% of households had at least one HIV-positive member, compared to 12.3% in rural settings^2^. About 22% of the 4,500 new HIV infections among persons aged 15 years and older in 2017 were adolescents^1^.

Adolescents are prone to HIV infection because of their involvement in risky sexual behavior such as unprotected, transactional, and cross-generational sex^3^, ^4^. About 25% of girls get pregnant before 18 years of age, which means that some adolescents have early and unprotected sex^5^. Due to the existing gaps in the prevention of mother-to-child HIV transmission (PMTCT) in Uganda, some children end up being vertically infected with HIV^6^,^7^. These children ultimately grow into adolescents who may become sexually active and spread HIV to their uninfected sexual partners^8^,^9^.

Despite the high vulnerability of adolescents to HIV due to risky sexual behaviors, efforts to address this issue are often hindered by cultural norms and communication barriers within families. In Ugandan culture, discussing sex-related issues with a child is generally considered taboo^10^. For that reason, many parents fear doing it^10^. They also lack age-appropriate, respectful, vocabulary, skills and information on how talk to their children about sexual and reproductive health^10^. That role is normally left to extended family members such as “Sengas” (sisters to fathers) and “Kojjas” (brothers to mothers), who in most cases do not live and rarely engage with the children^10^,^11^. Parents, especially fathers, are known to be strict and intimidating, which makes adolescents hide their sexual relationships from them and never talk about them at home^11^.

Overtime, there have been several state and community efforts, including the widely implemented Presidential Initiative on AIDS Strategy for Communication to Youth (PIASCY) which was launched in 2002 to address HIV/AIDS concern in Uganda holistically, targeting children, adolescents, school personnel, parents, and the wider community^12^. It was, however, mainly implemented in schools through child-centered activities such as assemblies, club meetings, music, dance, and drama as these provided a space for learners to discuss and reflect on their HIV/AIDS related concerns^12^. In 2018, the Ministry of Education and Sports (MOES) launched the national sexuality education framework, which gives a national direction for teaching children, adolescents, and young adults about their sexuality^13^. It focuses on ensuring that they get clear, current, age, and culturally appropriate information within the formal education system^13^. Despite these initiatives, some adolescents still indulge in early and unprotected sex^3^,^4^.

While previous studies in Uganda and other countries have examined HIV knowledge, its associated factors, and its relationship with sexual behavior, research findings have been inconsistent, and few have focused on adolescents aged 13-19 years^14^-^17^. Existing studies done in Belgium, Uganda, Nigeria, and Saudi Arabia have indicated that age, education, number of sexual partners, and history of testing for sexually transmitted infections (STIs) were positively associated with high levels of HIV knowledge^14^-^17^. In Iraq, research showed that male adolescents were significantly more likely to be knowledgeable about HIV compared to females^18^.

However, being knowledgeable about HIV does not always translate into practicing safe sex^19^. A study done in Brazil showed that there was no statistical association between knowledge on HIV/AIDS and sexual behavior among 14-19-year-old adolescents^19^. In contrast, a study in Nigeria found that the more knowledge adolescents had on HIV, the less involved they were in risky sexual behavior^4^. The average age of study participants in the Nigeria-based study was 17 years^4^.

This contradiction in findings points to a need to conduct more studies in other settings to confirm what the relationship between knowledge on HIV and sexual behavior truly is. In Uganda, a study conducted in Kampala found an association between HIV knowledge and sexual behavior among older youth (18-30 years)^15^. However, this association was only significant at the bivariate level of analysis and became non-significant at the multivariate level^15^. This therefore, leaves a critical gap in understanding whether similar patterns exist among adolescents.

To address this gap, our research study was conducted in an urban division located in Kampala, a district with a high HIV prevalence (6.9%) (2). We set out to assess the level of HIV knowledge among adolescents aged 13–19 years, identify factors associated with their knowledge, and examine the relationship between HIV knowledge and sexual behavior.

## Methods

### Study design, setting, and population

We conducted a cross-sectional study from May to July 2019 in an urban division in Kampala, Uganda. Kampala city consists of five administrative divisions. We selected one of those which had the highest number of areas with populations at high risk of contracting HIV such as male and female sex workers, and men who have sex with fellow men in Kampala according to results from a survey conducted in 2013^20^. The division comprises 22 parishes that are further subdivided into 118 zones. We enrolled 976 adolescents who were aged 13-19 years, had given written informed consent/assent to participate in the study, and were residing in 40 selected zones within the urban division during May 2019. We had planned to exclude all adolescents who were too ill to withstand study procedures, but based on this criterion, none were excluded.

### Sample size and sampling procedures

The sample size (N) was first computed using the formula for estimating a single mean of a characteristic because knowledge on HIV was a continuous variable. It was 348 based on estimates from a study done in Ghana^21^. Thereafter, (N) was computed using the formula for assessment of an association between a numerical predictor and a binary outcome. Based on results from the study done in Ghana, it was 439^21^. Finally, it was computed using the formula for the comparison of two means in two independent populations because sexual behavior was binary and HIV knowledge was continuous within each one of its categories. It was 230 based on estimates from a study done in Malaysia using the HIV Knowledge Questionnaire 18 - item version (HIV-KQ-18 scale)^22^. We decided to consider 439 as our study sample size to achieve all our specific study objectives. The Uganda-based study^15^, while similar to ours, did not provide several key estimates that were required for our sample size computations, hence the use of data from Ghana and Malaysia. We considered a 5% level of significance and 80% power. After adjusting for clustering and the possibility of a 10% non-response rate and a design effect (DE) of 2, our final sample size was 976 adolescents.

Cluster random sampling was used to select 9 out of the 22 parishes from the selected division, then 3-5 zones were randomly selected from each parish, bringing the total to 40 zones. With the help of Village Health Team (VHT) members who guided the researchers, we purposively selected an average of 25 households based on the presence of an eligible adolescent and willingness to participate from each zone. To enhance representativeness, the VHTs identified households from different areas within the zones that had varying socioeconomic status and residential settings. In case we found more than one eligible adolescent in a single household, only one was randomly selected to participate in the study. This was done by drawing lots using pieces of paper on which their name initials had been written.

### Ethical considerations

The study was approved by the Makerere University School of Medicine Research and Ethics Committee (SOMREC) (approval no. #REC REF 2019-078), and Kampala Capital City Authority (KCCA) permitted us to conduct the study within the selected division.

Written parental consent was elicited from parents/guardians who were found at home at the point of data collection of all 13-17-year-old adolescents using consent forms; assent was obtained from these children as well. Written informed consent was elicited from all 18 and 19-year-old adolescents and emancipated minors. Participants' questionnaires were allocated unique identification numbers to ensure that there was no possibility of linking the data collected to an individual; no names or any other personal identifying information was collected from the participants to ensure confidentiality.

### Data collection

We collected data using a single pretested interviewer-administered questionnaire that had three sections (socio-demographics, HIV knowledge, and sexual behavior). The HIV knowledge section was a modified version of the standard HIV Knowledge Questionnaire - 18 item version (HIV-KQ-18); The questionnaire contained 18 multiple-choice (“True”, “False” or “Don't know”) statements which were questions related to HIV transmission, prevention and treatment. Questions 3, 4, 12 and 17 from the original HIV-KQ-18 were about withdrawal during sexual intercourse, types of condoms, anal and oral sex (23); they were deemed vulgar and inappropriate for the adolescents by the investigators and were replaced with others shown in table II so that the tool could suit the Ugandan adolescent context. These changes were purely subjective and based on expert opinion. The modified version was pretested in another division that is very similar to where the study was implemented. Its scale reliability in the study population was 62.7%, which differed from that of the original instrument (75% - 89%) (23). It was also translated and back-translated into the local Luganda language. The minimum and maximum attainable scores from the HIV knowledge questionnaire for each participant were 0 and 18, respectively. A score of zero (0) meant that one was not knowledgeable about HIV at all, whereas 18 indicated that one was exceptionally knowledgeable.

The sexual behavior section assed if an adolescent had ever had sexual activity involving the insertion of the male penis inside the female vagina for sexual pleasure, reproduction or both. It also had a question assessing the number of people one had had sex with during the past 12 months and if they used a condom the last time they had it (this required a Yes/No response). Any participant who had had more than one sexual partner within the past 12 months or not used a condom the last time they had sex was categorized as having engaged in risky sexual behavior.

Data collection was strictly done from homes by 7 research assistants who were trained by the principal investigator on research ethics and use of the data collection tools. They gave community members information regarding what the study was about, elicited informed consent from whoever agreed to participate or have their child take part, and eventually asked eligible adolescents questions as guided by the questionnaire. They collected data on all days of the week to enable adolescents who were in school to participate in the study and were supervised by the principal investigator throughout the process. A Village Heath Team (VHT) member in each zone introduced the research team to community members every morning before the commencement of data collection. He/she also guided them on where to go as they collected the data.

### Data management and analysis

We analyzed data using Stata version 14.0. All data was analyzed as survey data because of the effect of clustering at parish and zonal levels. Descriptive statistics, like means and proportions, were used to describe the study population. Sums of the correct responses to the HIV-related questions for each participant were generated to reflect their knowledge on HIV. The HIV knowledge for the entire study population was summarized using a mean and standard deviation of the total scores for all the participants. The frequencies of the correct responses were examined, and their percentages were used to indicate the proportion of the study population that was knowledgeable about a specific aspect of HIV.

Sexual behavior was a binary outcome in this study; participants who reported that they had sex with someone that they were not committed to in a sexual relationship without using a condom and or had multiple sexual partners within 12 months before the study were categorized as having engaged in risky sexual behavior. Those that reported that they had sex with people they were committed to in sexual relationships or used various measures such as the use of condoms to avoid sexually transmitted infections in addition to those that were not sexually active were classified as having engaged in safe sexual behavior.

Multiple linear regression was used to assess the association between knowledge and the various predictors. There was no main predictor for that analysis. Binary logistic regression was used to assess the relationship between knowledge and sexual behavior. Knowledge on HIV was the main predictor. Testing of assumptions for suitability of the regression models was done for the regression analyses. All variables that had p-values < 0.2 at bivariate analysis were considered for multivariate analysis. A cut-off of 0.2 was chosen so that negatively confounded variables would not be excluded from the multivariate analysis. A stepwise assessment of variables for interaction and confounding was also done. A predictor was considered a confounder if it modified the effect of another on an outcome by > 10%. The level of significance was 0.05, and the confidence level was 95% throughout data analysis.

## Results

### Description of the study population

The mean age of the participants was 16 years (SD=2.05). About 9.1% (n=87) of the study population had engaged in risky sexual behavior. The mean age of sexual debut for both males and females was 16 years (SD=2.73). Majority of the participants in the study were females (54.6%, n = 533), and 98.4% had never been married (n = 960). Islam was the dominant religion (33.0%, n = 322), and close to three-quarters of the study population (72.5%, n = 708) were in school at the time the study was implemented. Most participants (36.2%, n=343) reported that they got information about HIV from science classes or clubs in schools. It was unlikely that participants used media platforms such as magazines, newspapers, radios, and the internet, including WhatsApp and Facebook, as a source of HIV related information. Table I summarizes the characteristics of the study population.

**Table I:** Characteristics of 976 adolescents from an urban division in Kampala city

Characteristic	Summary statistic
Age (years) mean (SD)	16 (2.05)
Safe sexual behavior n (%)	869 (90.9)
Age of sexual debut (years) mean (SD)	16 (2.73)
Female Gender n (%)	533(54.6)
**Marital Status n (%)**	
Never married	960(98.4)
Ever married	16(1.6)
**Religion n (%)**	
Muslim	322(33.0)
Catholic	268(27.5)
Pentecostal	199(20.4)
Protestant	169(17.4)
Pagans	2(0.2)
Others^a^	14(1.4)
**Current School Status n (%)**	
Currently in school	708(72.5)
Currently out of school	268(27.5)
**Alcohol consumption n (%)**	
Yes	37(3.8)
No	937(96.2)
**Use of recreational drugs n(%)^b^**	
Drug users	10(1.0)
Non-drug users	962(98.6)
**History of testing for HIV and knowledge of current HIV status**	
Never tested for HIV throughout life time	497(50.9)
Ever tested and know their current HIV status^c^	352(36.1)
Ever tested but don't know their current HIV status	127(13.0)
**Sources of HIV related information n (%)**	
Science classes/clubs in schools	343(36.2)
Television	188(19.8)
Health workers/Hospitals	132(13.9)
Parents	105(11.1)
Friends	97(10.2)
Others^d^	83(8.8)

SD Standard deviation

a Seventh Day Adventists, Baptists and Jehovah's Witnesses

b Cocaine, heroin or marijuana

c Knowledge of HIV status being current if any testing was done within 12 months before study

d Magazines, Newspapers, Radios and Internet including WhatsApp and Facebook

### Level of knowledge on HIV

The mean score on HIV knowledge for the entire study population was 11.30 (SD=2.91). None of the study participants scored zero (0), and only one scored 18. Of all the girls in the study, 33.8% reported that females cannot get HIV if they have sex during their periods. Table II summarizes scores on HIV related questions for the entire study population. Concerning the different sections of assessment of HIV knowledge, 68.1% (n=665) of all the 976 participants answered half or more of the HIV transmission-related questions correctly. Majority (86.9%, n= 848) answered half or more of the HIV prevention-related questions correctly, and 69.5% (n=678) participants answered 50% of the questions related to general knowledge on HIV and its treatment correctly.

**Table II:** Scores on HIV related questions for 976 adolescents from an urban division in Kampala city

Statement N=976	Correct responses (%)	Wrong responses (%)	No response (%)
1) Coughing and sneezing DO NOT spread HIV	43.0	49.0	8.0
2) A person can get HIV by sharing a glass of water with someone who has HIV	67.7	27.7	4.5
3) Using a condom reduces the risk of HIV transmission	85.8	9.1	4.8
4) A person can get HIV infection from mosquito bites	69.9	22.0	7.6
5) Bathing or washing one's private parts after sex protects a person from getting HIV	55.3	21.9	22.5
6) There is medication that can prevent pregnant women who are infected with HIV from having babies born with AIDS	78.1	10.0	11.5
7) A healthy-looking person can have HIV infection	89.9	6.3	3.0
8) There is a vaccine that can stop people from getting HIV	54.3	29.7	15.9
9) People are likely to get HIV by deep kissing, putting their tongue in their partner's mouth, if their partner has HIV	40.6	45.5	13.4
10) A girl/woman cannot get HIV if she has sex during her periods	40.7	33.8	25.0
11) There is a female condom that can help to decrease a woman's chances of getting HIV	70.5	12.5	16.7
12) There is a cure for HIV	66.2	24.8	8.6
13) A person cannot get HIV if he/she is on medication for a disease that is not AIDS like cough, malaria etc.	61.0	24.9	14.0
14) Having sex with more than one sexual partner can increase a person's chances of being infected with HIV	83.6	10.9	4.9
15) Taking an HIV test one week after having sex will tell a person if he/she has HIV	24.4	61.9	13.5
16) A person can get HIV if they share a toilet or bathroom with someone who has HIV	73.7	19.5	6.6
17) One can protect him/herself from HIV by abstaining from sex	84.2	12.3	3.3
18) Using Vaseline or baby oil with condoms lowers the chance of getting HIV	41.1	25.2	33.5

### Factors associated with knowledge on HIV

Age, gender, religion, and current school status were the only socio-demographic factors that were independently associated with HIV knowledge at bivariate analysis. Similarly, the family and lifestyle related factors: presence of parents, current involvement in sports club activities, and access to media also had associations with knowledge at that level of analysis. All those variables, in addition to the history of testing for HIV and knowledge of current HIV status, source of HIV related information, and being in an intimate relationship with a member of the opposite sex, were considered for multivariate analysis.

The final model was adjusted for gender and current involvement in sports activities. Age, current school status, history of HIV testing, and current knowledge of status were the factors that were significantly associated with HIV knowledge in the adjusted model. For every single year increase in age, an adolescent's score on knowledge on HIV was likely to increase by 0.365. An adolescent that was in school was likely to score 1.222 more in the HIV knowledge assessment test than one that was out of school.

Religion, the presence of parents, access to media sources of HIV related information, and being in an intimate relationship with a member of the opposite sex, despite being significant at bivariate analysis, did not have any significant effect on HIV knowledge in the presence of other variables. Tables III and IV summarize the factors associated with knowledge on HIV among all study participants.

**Table III:** Association between socio-demographic, family factors and HIV knowledge among adolescents from an urban division in Kampala city

Variable	N	Crudeβ [95% CI]	P value	Adjustedβ [95% CI]
**Age**	**976**	**0.269 [0.17 - 0.37]**	**<0.001**	**0.365 [0.24 - 0.49]**
**Gender**				
Male	443	Ref		Ref
Female	533	-0.558 [-1.04 - -0.08]	0.027	-0.307 [-0.78 - 0.17]
**Marital status**				
Never married	960	Ref		
Ever married	16	-0.494 [-2.09 - 1.10]	0.496	
**Religion**				
Christians	650	Ref		
Muslims	322	-0.325 [-0.69 - 0.04]	0.075	
**Current school status**				
Currently in school	708	Ref		Ref
**Currently out of school**	**268**	**-0.570 [-1.10 - -0.04]**	**0.038**	**-1.222 [-1.83 - -0.62]**
**Presence of parents**				
Both	806	Ref		
Mother alone	118	0.207 [-0.54 - 0.95]	0.540	
Father alone	32	-0.216 [-1.56 - 1.13]	0.720	
None	19	-1.310 [-3.13 - 0.51]	0.135	
**Who the child stays with**				
Parent(s)	679	Ref		
Relatives	212	-0.198 [ -0.89 - 0.49]	0.525	
Others^a^	82	-0.105 [-0.86 - 0.65]	0.757	

a Spouses, friends, employers or living alone

β Coefficient

CI Confidence interval

**Table IV:** Association between lifestyle, HIV and sexuality related factors and HIV knowledge among adolescents from an urban division in Kampala city

Variable	N	Crudeβ [95% CI]	P value	Adjustedβ [95% CI]	P value
**Involvement in sports club activities**					
Yes	551	Ref		Ref	
No	407	-0.584 [-0.94 - -0.23]	0.005	-0.387 [-0.79 - 0.01]	0.056
**Access to media**					
Yes	914	Ref			
No	42	-0.714 [-1.88 - 0.45]	0.194		
**History of HIV testing and knowledge of current HIV status**					
Never tested for HIV	497	Ref		Ref	
Ever tested and know their current HIV status	352	0.481 [-0.17 - 1.13]	0.125	0.148 [-0.51 - 0.81]	0.618
**Ever tested but don't know their current status**	**127**	**1.051 [0.37 - 1.73]**	**0.007**	**0.868 [0.20 - 1.53]**	**0.017**
**Source of HIV related information**					
Parent(s)	105	Ref			
Friends	97	0.507 [-0.27 - 1.29]	0.173		
Science clubs / classes in school	343	0.579 [-0.11 - 1.27]	0.090		
Health workers	132	0.663 [-0.29 - 1.62]	0.147		
Television	188	-0.418 [-1.16 - 0.32]	0.227		
Others^a^	83	0.138 [-0.74 - 1.02]	0.727		
**Being in an intimate relationship with a member of the opposite sex**					
Yes	220	Ref			
No	754	-0.260 [-0.66 - 0.14]	0.176		
**Past sexual experience**					
Ever had sex	256	Ref			
Never had sex	719	-0.175 [-0.58 - 0.23]	0.344		
**Number of sexual partners in the past 12 months**	976	0.036 [ -0.04 - 0.11]	0.314		

a Magazines, Newspapers, Radios and Internet including WhatsApp and Facebook, β Coefficient, CI Confidence interval

### Association between the level of knowledge on HIV and risky sexual behavior

At bivariate analysis, age, gender, marital status and current school status were the socio-demographic factors that had p-values less than 0.2 for their associations with risky sexual behavior. In addition to those, the family and lifestyle related factors: presence of parents, who the adolescent stayed with, alcohol consumption, and the use of recreational drugs were also independently associated with risky sexual behavior. Regarding HIV and sexuality related factors, history of testing for HIV and knowledge of current HIV status and being in an intimate relationship with a member of the opposite sex also had associations with risky sexual behavior at bivariate analysis and were therefore considered for multivariate analysis.

No matter an adolescent's score on knowledge on HIV, their odds of risky sexual behavior did not change in any significant way both independently and in the presence of other variables. Gender, current school status, alcohol consumption, use of recreational drugs, and being in an intimate relationship with a member of the opposite sex were associated with risky sexual behavior in the adjusted model that had HIV knowledge as a main predictor. The odds of risky sexual behavior among female adolescents were 0.61 less than those among their male counterparts. The odds of risky sexual behavior among adolescents that were out of school were close to 4 times those among those that were in school at the time the study was implemented.

Age, marital status, presence of parents, who the child stayed with, history of testing for HIV, and knowledge of current HIV status did not have any significant effect on risky sexual behavior. Tables V and VI summarize the results of the multivariate analysis for the association between HIV knowledge and sexual behavior among all study participants.

**Table V:** Association between socio-demographic, HIV and sexuality related factors and risky sexual behavior among adolescents from an urban division in Kampala city

Variable	Risky Sexual Behavior					
	Yes n(%)	No n(%)	Crude OR [95% CI]	P value	Adjusted OR [95% CI]	P value
**Knowledge on HIV**	87(9.1)	869(90.9)	0.99 [0.91 - 1.07]	0.689	0.97 [0.87 - 1.08]	0.565
**Age**	87(9.1)	869(90.9)	1.48 [1.28 - 1.71]	<0.001		
**Gender**						
Male	60(13.9)	372(86.1)	Ref		Ref	
**Female**	**27(5.2)**	**497(94.8)**	**0.34 [0.20 - 0.56]**	**0.001**	**0.39 [0.22 - 0.67]**	**0.004**
**Marital Status**						
Never married	83(8.8)	859(91.2)	Ref			
Ever married	4(28.6)	10(71.4)	4.14 [1.12 - 15.28]	0.036		
**Current school status**						
Currently in school	32(4.6)	669(95.4)	Ref		Ref	
**Currently out of school History of HIV testing and knowledge of current HIV status**	**55(21.6)**	**200(78.4)**	**5.75 [3.48 - 9.51]**	**<0.001**	**3.82 [2.02 - 7.22]**	**0.001**
Ever tested and know their current HIV status	46(13.4)	297(86.6)	Ref			
Never tested and ever tested for HIV but don't know current HIV status	41(6.7)	572(93.3)	0.46 [ 0.26 - 0.82]	0.015		
**Being in an intimate relationship with a member of the opposite sex**						
Yes	48(23.6)	155(76.4)	Ref		Ref	
**No**	**39(5.2)**	**713(94.8)**	**0.18 [0.09 - 0.34]**	**<0.001**	**0.33 [0.13 - 0.84]**	**0.025**

OR Odds ratio, CI Confidence interval

**Table VI:** Association between family and lifestyle related factors and risky sexual behavior among adolescents from an urban division in Kampala city

Variable	Risky Sexual Behavior					
	Yes n(%)	No n(%)	Crude OR [95% CI]	P value	Adjusted OR [95% CI]	P value
**Presence of parents**						
Both parents alive	65(8.2)	726(91.8)	Ref			
Only one parent alive or both parents deceased	22(13.4)	142(86.6)	1.73 [0.89 -3.36]	0.093		
**Who the adolescent stays with**						
Stays with parent(s)	41(6.1)	626(93.9)	Ref			
Others^a^	45(15.7)	241(84.3)	2.85 [1.77 - 4.60]	0.001		
**Alcohol consumption**						
Yes	14(42.4)	19(57.6)	Ref		Ref	
**No**	**72(7.8)**	**849(92.2)**	**0.12 [0.05 - 0.25]**	**<0.001**	**0.23 [0.09 - 0.56]**	**0.005**
**Use of recreational drugs^b^**						
Drug users	5(50.0)	5(50.0)	Ref		Ref	
**Non-drug users**	**81(8.6)**	**861(91.4)**	**0.09 [0.02 - 0.40]**	**0.005**	**0.17 [0.03 - 0.95]**	**0.044**
**Current involvement in sports club activities**						
Yes	46(8.5)	498(91.5)	Ref			
No	37(9.4)	357(90.6)	1.12 [0.55 - 2.31]	0.722		
**Access to media**						
Yes	79(8.83)	816(91.2)	Ref			
No	6(14.6)	35(85.4)	1.77 [0.63 - 4.96]	0.237		

a Spouses, friends, employers or living alone

b Cocaine, heroin or marijuana, OR Odds ratio, CI Confidence interval

## Discussion

This study provided important insights regarding knowledge on HIV amongst 13 to 19-year-old adolescents living in the selected division. All of the participants had at least some knowledge on HIV, as none scored zero (0) out of 18. Only one adolescent was exceptionally knowledgeable about HIV, scoring the maximum score of 18. The level of knowledge on HIV was generally moderate.

This was similar to findings from a study conducted in Malaysia^24^. Considering the fact that in Uganda, the Presidential Initiative on AIDS Strategy for Communication to Youth (PIASCY) was mainly implemented in schools, leaving many children that were out of school less informed about HIV, this moderate level of knowledge may be attributed to insufficient HIV related information received by the adolescents^12^. In addition to that, culturally, adults still withhold sex education (including that about HIV) from young family members^10^. The results indicated that the adolescents were more knowledgeable about HIV prevention than transmission. A similar finding was reported in a study done among adolescents in Belgium^14^. This finding is worrisome because, as evidenced in Brazil, a lack of clarity on modes of HIV transmission may become an element of vulnerability to HIV infection for young people^19^.

Some misconceptions concerning transmission, prevention, and treatment of HIV were observed in this study. Similar misconceptions about transmission of HIV through mosquito bites and sharing of toilets/bathrooms were observed in Nigeria, Cameroon, and Saudi Arabia^16^,^25^,^26^.

Our results indicated that knowledge on HIV increases with age. This finding is in accordance with previous research done in Belgium and Uganda^14^,^15^. This could be attributed to the fact that as one grows, they accumulate more knowledge about everything over the years. Adolescents that were still in school had more knowledge on HIV than those that were out of school. Previous studies done in Nigeria and Saudi Arabia have shown that education attainment is positively associated with HIV knowledge^16^,^17^. Male adolescents had higher scores on HIV knowledge than their female counterparts. This is consistent with what was reported in a study done among adolescents in Iraq but is different from those done in Cameroon and Malaysia, where gender did not have any effect on HIV knowledge^18^,^24^,^25^. Religion was independently associated with knowledge on HIV but in the presence of other variables, it had no association with HIV knowledge. Marital status was not in any way associated with HIV knowledge. This is consistent with the findings from Belgium and Uganda, where marital status and religion did not influence one's knowledge on HIV^14^,^15^.

Recent studies have not considered adolescents' involvement in sports club activities in investigating factors associated with HIV knowledge, but this study showed that those who were involved in any form of sports club activity had higher scores on HIV knowledge compared to those that were not involved in any. This may be because some HIV/AIDS programs specifically target youth hangouts such as sports activities^27^. Who the child stayed with did not in any way affect their knowledge on HIV.

This differs from what was reported in a study done in Uganda, where participants who stayed with their parents scored significantly lower scores on knowledge than those who did not stay with them^15^. Adolescents that had ever tested for HIV before participating in this study were more knowledgeable about HIV than those that had never tested. This may be because whenever they go to test for HIV, they are given general information about it. This finding is similar to what was reported among Belgian adolescents where higher scores on HIV knowledge were observed among participants who had tested for STIs in the past than those who had never tested^14^. At bivariate analysis, the source of HIV related information and being in an intimate relationship with a member of the opposite sex were associated with HIV knowledge. However, they were not associated with it at multivariate analysis. This was different from what was reported in Belgium, where adolescents who were in a relationship had significantly higher scores on HIV knowledge compared to those who were not^14^. Past sexual experience and number of sexual partners in the past 12 months were not associated with HIV knowledge. This is different from what was reported in Belgium where adolescents that had more than one sexual partner were more knowledgeable about HIV than those that had only one^14^.

Being knowledgeable about HIV did not mean that one engaged in safe sex. This result is consistent with what was reported in Brazil but differs from what was found in Nigeria, where those with high levels of knowledge on HIV were less likely to engage in risky sexual behavior^4^,^19^. This finding could be attributed to the fact that adolescents generally have a low perception of risk for getting infected with HIV and therefore no matter how much knowledge they accumulate on HIV, the may not feel the need to protect themselves^28^. Adolescence is also a stage characterized by excitement, curiosity, and the need to experiment sexually, so it is possible that in the moments when they are presented with an opportunity to have sexual intercourse, the excitement precedes what they know about HIV^29^,^30^.

The results of this study also indicated that male adolescents were much more likely to engage in risky sexual behaviors compared to females (OR = 0.39, p-value =0.004). This finding is not in any way different from what was reported in Jos Plateau state - Nigeria but differs from that reported in the one done in Makerere University - Uganda where male students were more likely not to engage in risky sexual behaviors compared to the females^4^,^15^. Adolescents who were out of school were 3.8 times more likely to engage in risky sexual behaviors compared to those who were in school. A previous study done in Uganda indicated that education attainment is positively associated with low risk in sexual behavior^15^. Therefore, the longer an adolescent is kept out of school, the more the risk of them being involved in risky sexual behavior is increased. Adolescents that either drank alcohol or used recreational drugs were more likely to engage in risky sexual behaviors cmpared to those that did not take any substance of that kind. This may be because of the impaired judgment that accompanies the misuse of alcohol and recreational drugs, which often results in the adolescents making poor decisions concerning their sexual relations. A meta-analysis indicated that there is indeed an association between alcohol/drug use and sexual behavior among adolescents^31^. The study results also indicated that adolescents who were in an intimate relationship with a member of the opposite sex were more likely to engage in risky sexual behavior compared to those who were not.

## Limitations of the study

The HIV knowledge assessment tool was not very reliable. Its value of Cronbach's alpha (62.7%) was less than the recommended 70% for good scale reliability. Nonetheless, the results from its assessment still gave us an insight into what adolescents know about HIV. A selection bias may have been introduced by selecting only one adolescent per household where multiple were eligible. This approach excluded siblings who may have shared similar risk factors, potentially influencing the results related to associated factors. The data collection tool had some very sensitive questions about sexual behavior, so the investigators had to rely on the participants' honesty and transparency. Sexual behavior was based solely on participants' self-reports, which might have been subject to perceived social desirability and recall bias. No validation of self-reported responses was conducted, and triangulation with other data sources, such as medical records, was not feasible in our study. This may have affected the accuracy of our estimation of the outcomes. Information about education level, a variable that is associated with HIV knowledge, was very limited in this study; all that was assessed was if an adolescent was in or out of school. A more detailed breakdown of the education levels attained by the participants would have given a more meaningful understanding of the effect of one's education status on their level of knowledge on HIV. The study design used is not the best in determining temporal relationships between the various variables. The generalizability of the study findings is limited to peri-urban settings. Refusal to participate was not documented in the study. A few adolescents were not interested in the research and therefore refused to participate. Quite a number of parents refused their children to participate out of fear that they were too young to be asked about sexual intercourse, but the numbers weren't recorded.

## Conclusions

Our study findings could serve as a baseline for HIV knowledge and sexual behavior assessment among 13-19-year-old adolescents for any investigators that might want to conduct a post-COVID-19 pandemic evaluation. Adolescents living in the selected urban division generally had a moderate level of knowledge on HIV. There are still misconceptions regarding HIV transmission, prevention, and treatment among these adolescents. The study showed evidence of the existence of associations between HIV knowledge and age, current school status, gender and one's current involvement in sports activities, history of HIV testing, and current knowledge of HIV status among 13 to 19-year-old adolescents. The study findings, particularly regarding adolescents' involvement in sports club activities and HIV knowledge, could be a basis for further research into using that avenue as a means to educate them about HIV and other sexual and reproductive health issues. Knowledge on HIV was not associated with risky sexual behavior. Being out of school, alcohol consumption, use of recreational drugs, and being in an intimate relationship with a member of the opposite sex significantly increased the adolescents' possibility to engage in risky sexual behavior.

There is, therefore, a need to continuously sensitize adolescents on the reality of HIV, its transmission, prevention, and consequences of infection. Adolescents should be encouraged to adopt safe sexual behavior. Their current sexual behaviors and those they will adopt and live by as they grow have a bearing on our progress in dealing with the HIV pandemic. The implementers of the national sexuality education framework need to design and implement more innovative programs that can address alcohol and recreational drug use, which drive young people into making poor decisions concerning their sexuality.
